# World Report: A Snapshot of Orthopaedic Surgery in The Netherlands

**DOI:** 10.2106/JBJS.25.00919

**Published:** 2025-10-07

**Authors:** Rudolf W. Poolman, Jacob J. Caron, Sebastiaan A.W. van de Groes, Cornelis Leendert Pieter van de Ree, Bart G. Pijls

**Affiliations:** 1 Department of Orthopaedic Surgery, JointResearch, OLVG, Amsterdam, The Netherlands; 2 Department of Orthopaedic Surgery, Leiden University Medical Center, Leiden, The Netherlands; 3 Royal Netherlands Orthopaedic Association, Amsterdam, The Netherlands; 4 Science Portfolio, Royal Netherlands Orthopaedic Association, ’s-Hertogenbosch, The Netherlands; 5 CORE (Consortium Orthopaedic REsearch), Dutch Orthopaedic Association, ’s-Hertogenbosch, The Netherlands; 6 Department of Orthopaedic Surgery, Erasmus MC, Rotterdam, The Netherlands

## Introduction: A Collaborative and Data-Driven Community

The Netherlands, a small European country with >17 million inhabitants, has a highly organized and collaborative orthopaedic community. Overseen by the Royal Netherlands Orthopaedic Association (Koninklijke Nederlandse Orthopaedische Vereniging [NOV]), approximately 850 orthopaedic surgeons and 300 residents in training provide care across a network of university medical centers, teaching hospitals, and private clinics (Table 1). The defining characteristic of the Dutch orthopaedic landscape in recent years has been a collective drive toward value-based health care, underpinned by a robust data infrastructure and a commitment to the principles of “gepast gebruik,” or appropriate use of care^1^. This report provides a snapshot of the key initiatives, challenges, and opportunities that define modern orthopaedic surgery in The Netherlands.

**Table 1.** Key Insights

The Dutch orthopaedic community, comprising approximately 850 surgeons and 300 residents, is characterized by a strong collaborative culture both nationally and internationally and a national focus on value-based health care and appropriate use of care.
A systematic circle of appropriate use, encompassing agenda setting, evaluation, and implementation, guides national research and quality improvement efforts, aiming to reduce research waste and enhance patient outcomes.
The Dutch Arthroplasty Register (LROI), the third-largest worldwide, is a cornerstone of quality assurance and a powerful engine for innovation, driving studies on implant performance, patient outcomes, and predictive modeling.
Current innovations focus on enriching the LROI’s capabilities through projects linking hospital data, measuring care complexity in revisions, and creating platform-trials for flexible intervention evaluations, thereby moving from a reactive to a proactive registry.
Key challenges include managing an aging population and rising health-care costs, although opportunities lie in leveraging the robust data infrastructure and collaborative mindset to further personalize patient care and implement evidence-based practices efficiently.

## The Circle of Appropriate Use: From Question to Practice

A central philosophy in Dutch orthopaedics is the “circle of appropriate use” (Table 1). This systematic, 3-step approach ensures that clinical practice is continuously questioned, evaluated, and improved on the basis of scientific evidence and patient values. This cycle is our national strategy to reduce research waste and ensure that innovations truly benefit patients.

### Setting the Agenda: Identifying What We Do Not Yet Know

The first step is to identify and prioritize evidence uncertainties^2^. Rather than relying on individual interests, the Dutch orthopaedic community has established a formal process to create a national Health Research Agenda. In a multi-stakeholder process involving surgeons, patients, and other health-care professionals, evidence gaps in daily practice are systematically collected. Through a modified Delphi consensus method, these uncertainties are ranked, resulting in a widely supported Health Research Agenda. This process ensures that research funding and effort are directed toward the most pressing clinical questions, such as identifying the most effective treatments for common conditions or optimizing care pathways for specific patient groups. The Consortium Orthopaedic REsearch (CORE) is the network for initiating and continuing Dutch scientific research within the NOV.

### Evaluating: Rigorous Assessment of Interventions

Once a key uncertainty is identified, the next step is rigorous evaluation, often through nationally funded, multicenter, randomized controlled trials (RCTs). A prime example is the ESCAPE trial, which compared arthroscopic partial meniscectomy with physical therapy for degenerative meniscal tears. The 5-year follow-up results demonstrated that physical therapy was noninferior to surgery, leading to a substantial shift in national practice and de-implementation of a low-value intervention^3^. Another key study, the FRAIL-HIP study, evaluated operative treatment compared with nonoperative management for frail, institutionalized patients with hip fractures. It concluded that, for this specific, vulnerable population, nonoperative management, chosen through shared decision-making, is a viable option—thus challenging the surgical default^4^. These large-scale, pragmatic trials are a hallmark of the Dutch research environment, providing real-world evidence to guide clinical decisions.

### Implementing: Translating Evidence into Care

The final, and often most challenging, step is implementing evidence into daily practice. The findings from evaluation studies are disseminated through the NOV and are integrated into national guidelines^5^. The Dutch Arthroplasty Register (Landelijke Registratie Orthopedische Interventies [LROI]) plays a crucial role here by monitoring practice patterns. For instance, following the ESCAPE trial^6^, a measurable decrease in meniscectomies for degenerative tears can be observed. This feedback loop, in which evidence leads to new guidelines and registry data monitor their uptake, is fundamental to closing the circle and realizing a continuously learning health-care system.

## LROI: The Engine of Quality and Innovation

Established in 2007, the LROI has become a cornerstone of Dutch orthopaedic practice with a completeness of >98% and including >1.2 million joint replacements^7^. Its primary function is quality assurance through monitoring the performance of prostheses and identifying outliers (Table 1). However, its value extends far beyond that. The LROI allows case identification in the event of a product recall or adverse surgical outcomes, it provides insight into practice variation, and it serves as a powerful database for scientific research, enabling studies on patient-reported outcomes, risk factors for revision, and the effectiveness of different surgical approaches. For example, the LROI data have been instrumental in studies assessing whether machine learning models can outperform traditional regression for predicting revision surgery, concluding that, for now, their predictive value remains limited in this context^8^. To expand these opportunities, the LROI is now also open for the registration of interventions other than joint arthroplasties.

## LROI to the Next Level 2025

To maintain its leading position and further enhance its utility, the LROI has initiated the “LROI to the Next Level 2025” program. This call for innovation (Table 1) has resulted in the funding of 3 promising projects set to transform the registry:

1. Next Level via Datapoort: Led by Professor Job N. Doornberg, this project will establish a direct, automated link between hospital electronic health records and the LROI. This will enrich the registry with more detailed clinical data, reduce the administrative burden of manual data entry, and create a more comprehensive data set for research.
2. Proactive LROI with a Care Complexity Meter: Dr. José Smolders is leading a project to implement a validated tool to measure the complexity of revision arthroplasty cases. This will allow for fairer comparisons of hospital and surgeon performance by adjusting for case mix, transforming the LROI from a reactive signal detector to a proactive, nuanced quality instrument.
3. LROI INNOVATE Platform: Led by Professor Rudolf W. Poolman, this project will develop a platform trial for flexible and efficient evaluation of interventions within the LROI infrastructure. This “trials-within-a-registry” framework will make it easier and cheaper to conduct pragmatic experiments on new implants, techniques, or care pathways, accelerating the evaluation cycle.

## From Evidence-Based Surgery to Societal Impact

**Fig. 1:**
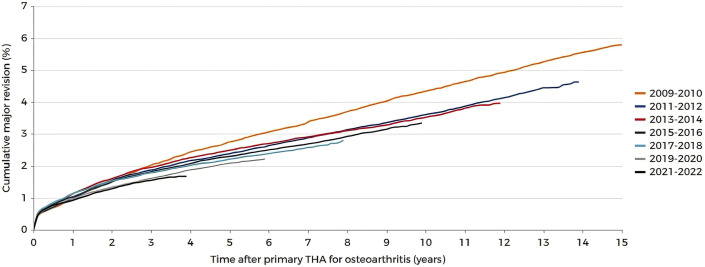
Kaplan-Meier graph showing the cumulative major revision percentage after total hip arthroplasty (THA) for primary osteoarthritis by procedure year. (With permission from LROI, 2025 Annual report. Retrieved from https://www.lroi.nl/jaarrapportage/hip/total-hip-arthroplasty/survival [accessed 2025 Aug 26].)

**Fig. 2:**
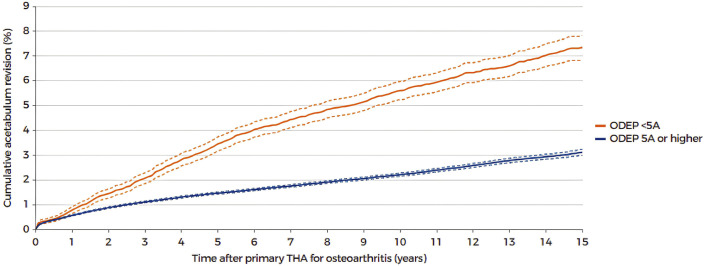
Kaplan-Meier graph showing the cumulative acetabular revision percentage with 95% confidence intervals after total hip arthroplasty (THA) for primary osteoarthritis by Orthopaedic Data Evaluation Panel (ODEP) rating. (With permission from LROI, 2024 Annual report. Retrieved from https://www.lroi.nl/jaarrapportage/hip/total-hip-arthroplasty/survival [accessed 2025 Aug 26].)

Regarding joint replacement, the focus of the Dutch orthopaedic community on evidence-based surgery has resulted in national decreases in revision rates after knee and hip joint replacements, as can be seen in Figure 1 for the hip. This focus includes national policies of routinely using implants that are rated 5A or higher by the Orthopaedic Data Evaluation Panel (ODEP), radiostereometric studies for early detection of unsafe implants, and registry studies for quality monitoring and post-market surveillance^9-14^. As can be seen from Figure 2, implants with a proven track record (ODEP 5A or higher) have substantially lower revision rates than implants without a proven track record. Taken together, this evidence-based approach has resulted in societal impact consisting of fewer revision surgeries for patients, reduced health-care cost for the national insurance, reduced carbon emissions, and less strain on the health-care system^15^.

## Challenges and Opportunities for the Future

The Dutch orthopaedic community faces challenges common to many Western health-care systems, including an aging population, the rising prevalence of osteoarthritis, pressure to control costs, and strain on the health-care system. A key internal challenge is ensuring the efficient implementation of evidence and reducing the evidence-practice gap.

The Dutch Arthroplasty Register (LROI), the third-largest worldwide, is a cornerstone of quality assurance and a powerful engine for innovation, driving studies on implant performance, patient outcomes, and predictive modeling.

However, the opportunities are substantial. The collaborative culture, the national commitment to appropriate care, and the unparalleled data infrastructure of the LROI and linked patient-reported outcomes data provide a unique ecosystem for innovation. The future of Dutch orthopaedics lies in leveraging these strengths to move toward a more personalized and predictive model of care. By combining clinical data such as registry data and patient-reported outcomes, we aim to choose not only the right treatment for the right patient group, but also the right treatment for each individual. The LROI to the Next Level projects are the first step in this exciting direction, promising a future in which our rich data sources are translated into even better outcomes for every patient whom we treat.
